# The Arginine Methyltransferase PRMT6 Cooperates with Polycomb Proteins in Regulating *HOXA* Gene Expression

**DOI:** 10.1371/journal.pone.0148892

**Published:** 2016-02-05

**Authors:** Claudia Stein, René Reiner Nötzold, Stefanie Riedl, Caroline Bouchard, Uta-Maria Bauer

**Affiliations:** Institute for Molecular Biology and Tumor Research (IMT), Philipps-University of Marburg, Marburg, Germany; University of St Andrews, UNITED KINGDOM

## Abstract

Protein arginine methyltransferase 6 (PRMT6) catalyses asymmetric dimethylation of histone H3 at arginine 2 (H3R2me2a), which has been shown to impede the deposition of histone H3 lysine 4 trimethylation (H3K4me3) by blocking the binding and activity of the MLL1 complex. Importantly, the genomic occurrence of H3R2me2a has been found to coincide with histone H3 lysine 27 trimethylation (H3K27me3), a repressive histone mark generated by the Polycomb repressive complex 2 (PRC2). Therefore, we investigate here a putative crosstalk between PRMT6- and PRC-mediated repression in a cellular model of neuronal differentiation. We show that PRMT6 and subunits of PRC2 as well as PRC1 are bound to the same regulatory regions of rostral *HOXA* genes and that they control the differentiation-associated activation of these genes. Furthermore, we find that PRMT6 interacts with subunits of PRC1 and PRC2 and that depletion of PRMT6 results in diminished PRC1/PRC2 and H3K27me3 occupancy and in increased H3K4me3 levels at these target genes. Taken together, our data uncover a novel, additional mechanism of how PRMT6 contributes to gene repression by cooperating with Polycomb proteins.

## Introduction

In mammals nine Protein Arginine Methyltransferases (PRMT1-9) have been identified, which transfer a methyl group from the ubiquitous cofactor S-adenosyl-L-methionine to the terminal guanidino nitrogens of arginine residues in target proteins. Subsequent to monomethyl-arginine, type I PRMTs form asymmetric (ω-N^G^,ω-N^G^) dimethyl-arginine, whereas type II enzymes give rise to symmetric (ω-N^G^,ω-N‘^G^) dimethyl-arginine [1]. Methylation of an arginine does not alter the positive charge of the guanidinium side chain, but changes its structure and the affinity between the substrate and its binding partners resulting in promotion or inhibition of interactions [2]. A multitude of nuclear and cytoplasmic proteins are substrates of arginine methylation, by which means PRMTs regulate a wide range of essential cellular functions [2].

PRMT6 was identified in 2002 by using the high sequence conservation of the PRMT catalytic domain for homology search in the human genome [3]. The enzyme belongs to the type I enzymes and prefers mono-methylated arginine as substrate [4,5]. In agreement with its primary nuclear localisation, PRMT6 methylates chromatin proteins. Similar to other PRMTs, PRMT6 typically targets glycin-arginine rich (GAR) motifs in substrates [6]. Accordingly, PRMT6 has been shown to modify HMGA1 and fibrillarin suggesting its involvement in chromatin architecture, splicing and rRNA processing [3,7,8]. However, PRMT6 methylates also non-GAR motifs, such as in the Tat protein of HIV1 leading to restriction of viral transcription and replication [9]. Automethylation of PRMT6 in a non-GAR motif results in increased protein stability and therefore activity [10]. Interestingly, PRMT6 was also reported to modify the DNA polymerase β thereby enhancing base excision repair [11] and the androgen receptor contributing to neurodegeneration [12]. Furthermore, PRMT6 acts as a histone methyltransferase on all four core histones, such as H3 at R2 [5,13], H2A at R29 [14] and H3 at R42 [15], thus leading to either transcriptional repression or activation. Due to transcriptional repression of tumor suppressor genes, PRMT6 promotes cell proliferation and blocks senescence [16–19].

The mechanism of transcriptional repression via asymmetric dimethylation of H3R2 (H3R2me2a) has intensively been studied. The H3R2me2a mark is a negative regulator of H3K4 trimethylation (H3K4me3), as it inhibits the activity of the H3K4 methyltransferase MLL1 and blocks recruitment of the MLL-complex subunit WDR5 to histone H3 [5,13,20]. Crystal structure analysis of WDR5 supports this antagonism between the two marks, as hydrogen bonding between the WD40 domain of WDR5 and the guanidino group of R2 is disturbed by asymmetric dimethylation [21,22]. Accordingly, overexpression of PRMT6 leads to global reduction of H3K4me3 levels and ChIP (chromatin immunoprecipitation) analysis showed a countercorrelation between the genomic occurrence of H3K4me3 and H3R2me2a [5,13]. Given that H3K4me3 generally marks the TSS (transcriptional start site) of actively transcribed as well as paused genes, reduction of the H3K4me3 levels by PRMT6-mediated H3R2me2a causes transcriptional repression, which has been demonstrated for a selection of PRMT6 target genes, such as *HOX* genes, MYC-target genes and *TSP-1* [5,13,20,23]. In agreement with this, H3R2me2a is not enriched at promoters of active genes and occurs at silent genomic regions, such as pericentromeric chromatin [24,25]. Apart from interfering with the deposition of H3K4me3, H3R2me2a might also impair the recognition of H3K4me3, as some readers of H3K4me3 are blocked by the presence of an adjacent H3R2me2a mark, for example the TFIID subunit TAF3 and the NURF subunit BPTF [20,26,27]. However, it is not clear to which extent H3K4me3 and H3R2me2a might coexist on the same histone tail.

Vice versa, the enzymatic activity of PRMT6 is influenced as well by neighbouring histone modifications, as the presence of the H3K4me3 mark reduces the ability of PRMT6 to modify R2 [5,13,20]. In contrast, premodification of H3 peptides with K27 di- and trimethylation (H3K27me2 / H3K27me3) enhances the methyltransferase activity of PRMT6 towards R2 [5]. Further, the genomic occurrence of H3R2me2a and H3K27me3 coincides at a distinct set of promoters, which are transcriptionally inactive [28–30]. H3K27me3 is a repressive histone mark, predominantly mediated by the EZH2 protein and deposited at facultative heterochromatin as well as silent genes, in particular differentiation-associated genes and tumor suppressor genes [31]. EZH2 together with the subunits EED, SUZ12 and RbAp48 comprises the canonical Polycomb repressive complex 2 (PRC2), which is able to associate with several other proteins allowing the formation of diverse complexes [31–34]. PRC2 cooperates with a second complex, the Polycomb repressive complex 1 (PRC1). PRC1 consists of four core subunits, which have several homologues in mammals giving rise to various complex combinations: CBX (chromobox) proteins, PH (Polyhomeotic) proteins, PSC (Posterior sex combs) proteins and SCE (Sex combs extra) proteins [35]. The SCE proteins, RING1A and RING1B, account for the enzymatic activity of PRC1 by mono-ubiquitylating H2A at K119. CBX-containing PRC1 complexes recognise the H3K27me3 mark deposited by PRC2 via the chromodomain of the CBX subunit. Together, PRC1 and PRC2 achieve the stable transcriptional repression of PRC target genes.

Given that both H3K27me3 and H3R2me2a are repressive histone marks and co-occur at a subset of gene promoters, we asked here whether PRMT6 influences PRC-mediated repression of differentiation-associated target genes. We find that in NT2/D1 cells, a model for neuronal differentiation, PRMT6 and subunits of PRC1 as well as PRC2 are bound to the same regulatory regions of rostral *HOXA* genes and diminish their transcriptional activation. Further, we show that PRMT6 interacts with subunits of the PRC1 and PRC2 complexes. Interestingly, depletion of PRMT6 results on the one hand in declined PRC1/PRC2 recruitment as well as H3K27me3 levels and on the other hand in increased H3K4me3 levels at the rostral *HOXA* genes. Taken together, our data uncover a novel, additional mechanism of how PRMT6 contributes to gene repression by cooperating with Polycomb proteins.

## Materials and Methods

### Cell lines, antibodies and plasmids

NT2/D1 and HEK293 cells were maintained in DMEM supplemented with 10% fetal calf serum (FCS, Gibco/BRL) at 37°C and 5% CO_2_. To induce neuronal differentiation of NT2/D1 cells with 0.1 μM ATRA (all-*trans* retinoic acid from Sigma) we followed the protocol described elsewhere [36].

The following antibodies were employed: rabbit affinity-purified anti-PRMT6 was produced using His-tagged proteins corresponding to amino acids 60–375 of human PRMT6; anti-H3 (05–499) from Upstate; anti-H3R2me2a (04–808), anti-H3K27me3 (07–449) and anti-H3K4me3 (07–473) from Millipore; anti-Myc (9E11), anti-GFP (G6539), anti-Flag (M2) (F3165), mouse IgG (I5381) and rabbit IgG (I5006) from Sigma; anti-CDK2 (sc-163) from Santa Cruz; anti-CBX8 (A300-882A) from Bethyl Laboratories; anti-HPH1 [37], anti-HPH2 [38], anti-BMI1 [39], anti-CBX8 [40], anti-EZH2 [41], anti-RING1A [42], anti-RING1B [37] were kindly provided. The expression construct for HPH1 (Rae28) is described in [43]. The expression construct of Myc-HPH2 was a gift from H. Koseki (RIKEN Yokahma Institute). The expression constructs for CBX2, CBX4 and CBX8 were published by [40]. Flag-EZH2 was a gift from D. Reinberg.

### Plasmid and siRNA transfection

Short interfering RNA (siRNA) oligonucleotide duplexes were obtained from Eurogentec or Dharmacon for targeting PRMT6, CBX8 and EZH2, respectively. The target sequences of siRNAs are listed in the Supporting Information (S1 Text). Transfections of siRNAs in NT2/D1 cells were performed with the aid of Lipofectamine RNAiMAX (Invitrogen) according to the manufacturer’s instructions. Plasmids were transfected using the PEI or calcium phosphate methods.

### Reverse transcription quantitative PCR (RT-qPCR) and chromatin immunoprecipitation quantitative PCR (ChIP-qPCR)

Total RNA was isolated using Seqlab RNA-Mini-Kit according to the protocol and applied (0.5–1 μg) to reverse transcription (RT) by incubation with oligo(dT)_17_ primer and M-MLV reverse transcriptase (Invitrogen), as recommended by the manufacturer. ChIP experiments were performed as described in [5]. The cDNA and eluted chromatin were subjected to qPCR analysis gene-specific primers listed in the Supporting Information (S1 Text). Quantitative PCR was performed using Absolute QPCR SYBR Green Mix (Thermo Scientific) and the Mx3000P real-time detection system (Agilent). For RT-qPCR *UBIQUITIN* gene transcription was used for normalisation. ChIP-qPCR results were calculated as % input. All experiments were performed at least three times and each experiment in triplicate. Reproducible and representative data sets are shown. Error bars represent mean +/- S.D. of triplicate reactions.

### Gel filtration analysis

To prepare whole-cell extracts, cells were lysed in IPH-buffer (150 mM NaCl, 5 mM EDTA, 0.5% (v/v) NP-40, 50 mM Tris, pH 8.0) including protease inhibitors and incubated 30 minutes at 4°C under rotation. DNA was digested using Benzonase for at least one hour at 4°C. Subsequently, proteins (3–5 mg) were separated on a Superose 6 HR 10/30 using the Äkta^TM^ Purifier 10 System (GE Healthcare) at a flow rate of 0.7 ml/minute and a pressure of 0.3–0.4 MPa. Six ml fractions were collected and either analysed by Western blot or used in co-immunoprecipitation followed by Western blot.

## Results

### PRMT6 and the PRC complexes occupy the same regulatory regions of *HOXA* genes during neuronal differentiation

**Fig 1:**
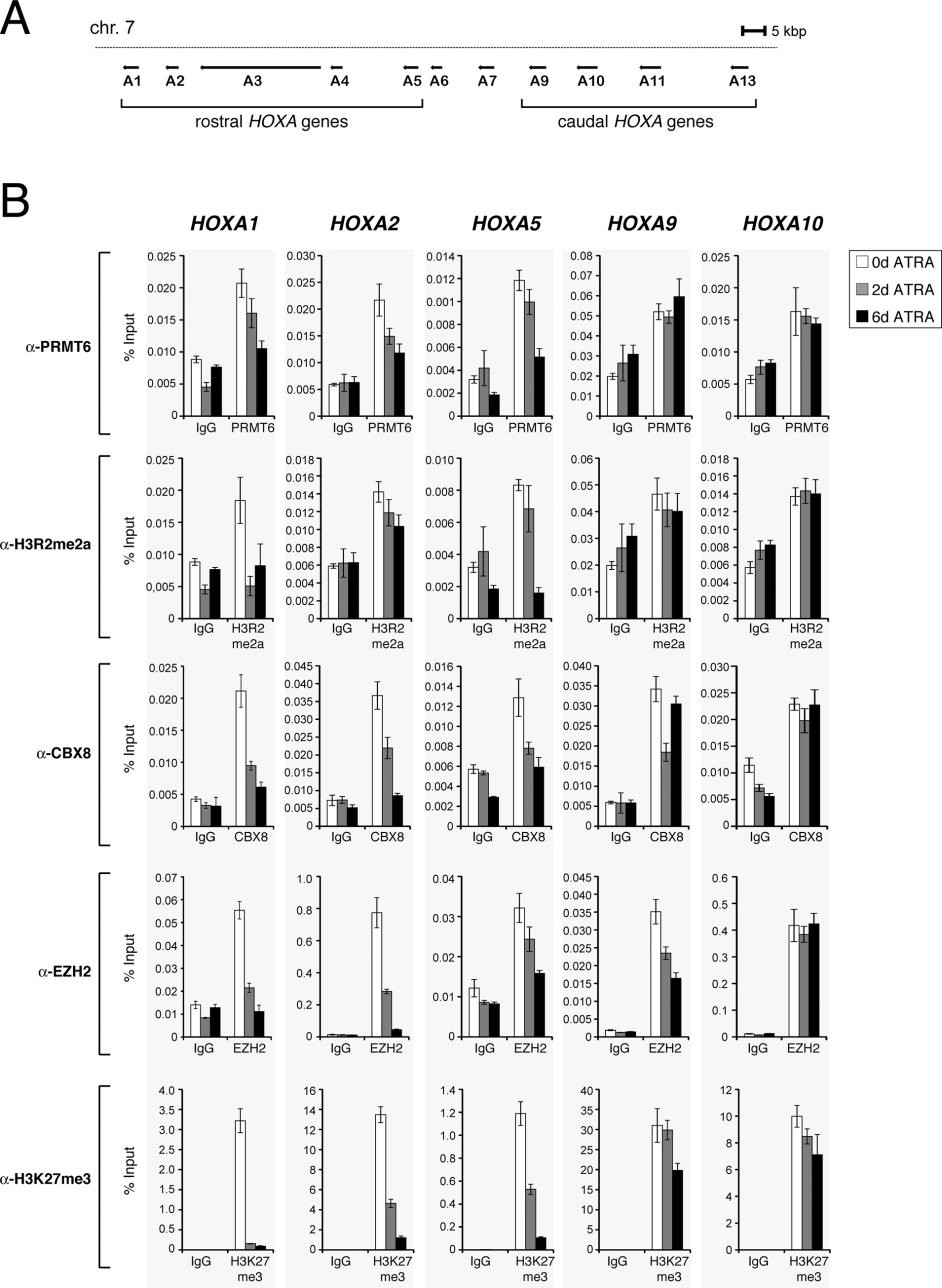
PRMT6 and subunits of PRC1/PRC2 co-occupy the HOXA gene cluster. (**A**) Schematic representation of the *HOXA* gene cluster consisting of 11 genes (*HOXA1-13*) and located on chromosome 7. According to their expression pattern, the 3’-located genes *HOXA1-5* belong to the rostral genes and the 5’-located *HOXA9-13* to the caudal genes. (**B**) NT2/D1 cells were left untreated (0 d) or treated for 2 days (2 d) and 6 days (6 d) with 0.1 μM ATRA. Subsequently, the cells were subjected to ChIP analysis using PRMT6-, CBX8- as well as EZH2-specific antibodies and antibodies recognising the H3R2me2a and H3K27me3 marks. Isotype-specific IgG served as control antibody. Immunoprecipitated DNA was analysed in triplicates by qPCR with primers for the *HOXA1*, *A2*, *A5*, *A9* and *A10* genes. Mean values were calculated as percentage input (% Input) and error bars represent mean +/- S.D. of the triplicates.

The human embryonal carcinoma (EC) cell line NT2/D1 represents a well-characterised model system to study the regulation of the *HOXA* gene cluster (Fig 1A for illustration of the *HOXA* cluster) [36,44–46]. Upon all-*trans* retinoic acid (ATRA) treatment NT2/D1 cells differentiate along the neuronal lineage, which is accompanied by transcriptional upregulation of rostral (3`) *HOXA* genes *HOXA1-5*, whereas no or only subtle changes in expression of caudal (5`) *HOXA* genes *HOXA9-13* have been observed [47,48]. These ATRA-induced transcriptional changes have been reported to be accompanied by a loss of PRC1 and PRC2 recruitment and a reduction of H3K27me3 levels at the rostral *HOXA* genes [36]. Importantly, the *HOXA2* gene is also a well-established target gene of PRMT6-mediated repression [5,20]. The finding that H3R2me2a and H3K27me3 coincide at human promoter regions [28] suggests a potential positive crosstalk between PRMT6- and Polycomb-mediated transcriptional repression. To further investigate PRMT6-occupied sites at *HOXA* genes and how these relate to the binding mode of PRC1 and PRC2 at the gene cluster, we employed ChIP (chromatin immunoprecipitation) analysis of undifferentiated, 2 and 6 days ATRA-differentiated NT2/D1 cells. We determined the occurrence of PRMT6, its histone modification H3R2me2a, CBX8 as a representative for PRC1, EZH2 as a representative for PRC2 and H3K27me3 at regulatory regions and promoters of various rostral *HOXA* genes, such as *HOXA1*, *A2* and *A5*, as well as the caudal *HOXA* genes, *HOXA9* and *A10*. We found that PRMT6 and H3R2me2a are present at the regulatory regions of all investigated *HOXA* genes in undifferentiated cells and that their occurrence remained unchanged at the *HOXA9* and *A10* gene promoters throughout the time-course of differentiation (Fig 1B). In contrast, at the rostral genes, *HOXA1*, *A2* and *A5*, their occurrence was successively lost during differentiation (Fig 1B). This recruitment pattern of PRMT6 and H3R2me2a at the different *HOXA* genes coincided with the binding of CBX8 and EZH2 and the occurrence of H3K27me3. Taken together, these data identify the *HOXA* gene locus as a common recruitment site for PRMT6, Polycomb proteins and their corresponding histone modifications in NT2/D1 cells.

### PRMT6 and PRC complexes repress the differentiation-associated transcriptional activation of rostral *HOXA* genes

**Fig 2:**
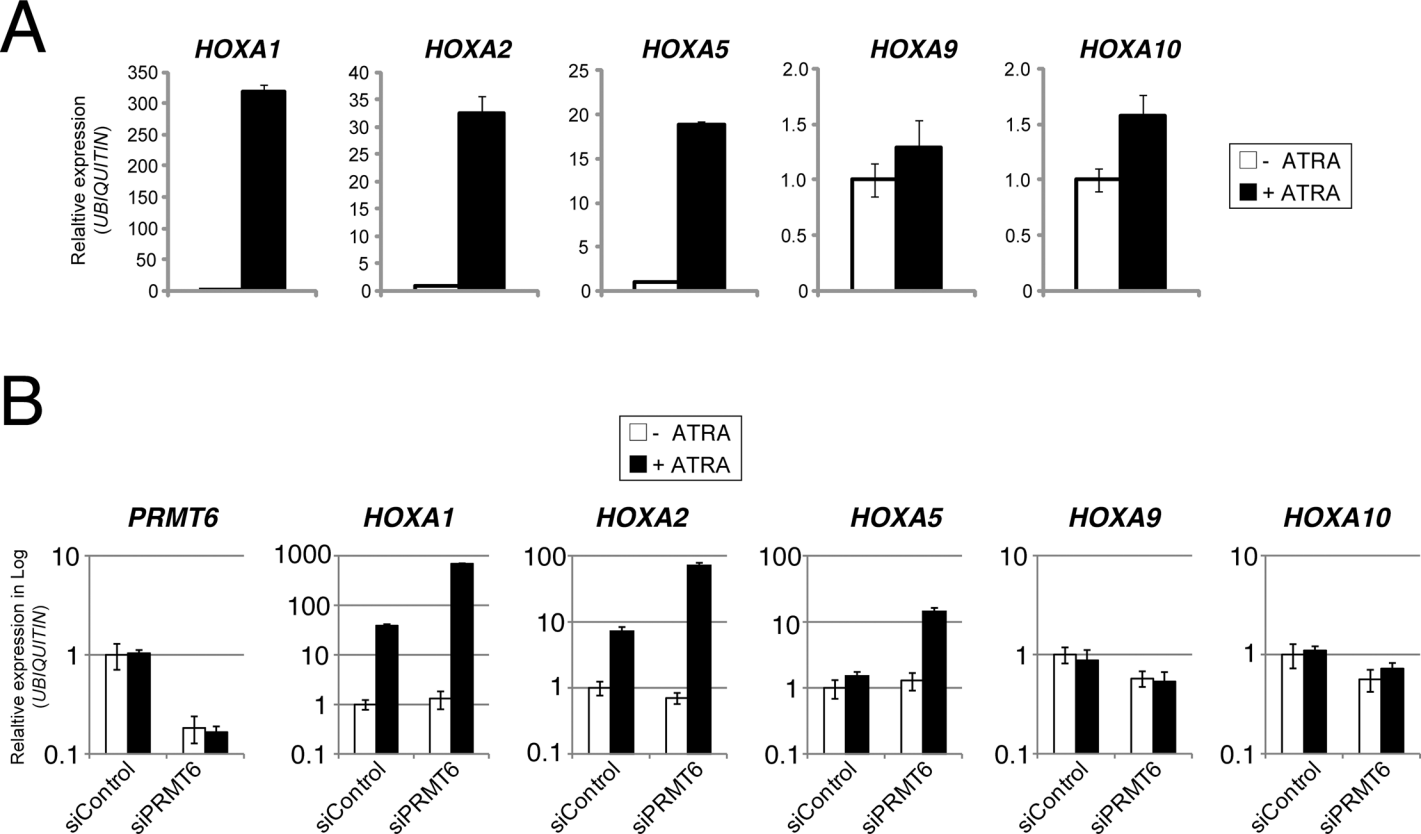
PRMT6 represses the ATRA-mediated transcriptional activation of rostral *HOXA* genes, but has no influence on the transcription of caudal *HOXA* genes. (**A**) NT2/D1 cells were left untreated (-) or treated for 2 days (+) with 0.1 μM ATRA. Subsequently, total RNA was prepared and analysed in triplicates by RT-qPCR for transcript levels of *HOXA1*, *A2*, *A5*, *A9* and *A10* normalised to *UBIQUITIN* transcription. Error bars represent mean +/- S.D. of the triplicates. Transcript levels of untreated cells were set to 1. (**B**) NT2/D1 cells were transfected with control siRNAs (siControl) or siRNA directed against PRMT6 (siPRMT6 = siPRMT6_1). Forty-eight hours post transfection cells were left untreated (-) or treated for 2 days (+) with 0.1 μM ATRA. Subsequently, total RNA was prepared and analysed in triplicates by RT-qPCR for transcript levels of *PRMT6*, *HOXA1*, *A2*, *A5*, *A9* and *A10* normalised to *UBIQUITIN* transcription. Error bars represent mean +/- S.D. of the triplicates. Transcript levels of untreated and siControl-transfected NT2/D1 cells were set to 1.

The genomic co-occurrence of PRMT6 and Polycomb proteins during neuronal differentiation of NT2/D1 cells suggests a potential positive crosstalk between the two repressive pathways in the transcriptional regulation of *HOXA* genes. We therefore aimed to investigate whether the presence of PRMT6 represses transcription of *HOXA* genes in NT2/D1 cells and how PRC complexes influence transcription of these genes. To this end, we initially confirmed by RT-qPCR the ATRA-driven transcriptional effects on rostral and caudal *HOXA* gene expression in NT2/D1 cells. As expected, upon ATRA treatment *HOXA1*, *A2* and *A5* genes were transcriptionally activated, whereas *HOXA9* and *A10* transcript levels remained unchanged (Fig 2A). Next, we established siRNA-mediated depletion of PRMT6 in these cells by transfection of siRNA targeting PRMT6 (siPRMT6) or control siRNA (siControl). Subsequently, RT-qPCR analysis was performed to measure transcript levels of *PRMT6* and the various *HOXA* genes in untreated as well as 2 days ATRA-treated NT2/D1 cells. Thereby, an efficient knockdown of *PRMT6* was observed and we further showed that transcript levels of *PRMT6* remain unchanged upon ATRA-induced differentiation in control cells (Fig 2B). Consistent with our observations in wild type NT2/D1 cells (Fig 2A), *HOXA1* and *A2* genes were transcriptionally induced (*HOXA5* weakly), while *HOXA9* and *A10* transcript levels remained constant upon ATRA treatment in siControl transfected cells (Fig 2B). Interestingly, depletion of PRMT6 did not significantly influence the transcriptional activity of the rostral as well as caudal *HOXA* genes in undifferentiated cells. However, it resulted in increased transcriptional activation of *HOXA1*, *A2* and *A5* genes upon ATRA treatment compared to control transfected cells, whereas *HOXA9* and *A10* transcript levels were not altered (Fig 2B). Furthermore, the observations for the rostral *HOXA* genes were confirmed using alternative siRNAs directed against PRMT6 (S1 Fig). Taken together, these data suggest that PRMT6 acts as a transcriptional repressor of rostral *HOXA* genes by fine-tuning the transcriptional activation of these genes in response to ATRA-induced neuronal differentiation of NT2/D1 cells.

**Fig 3:**
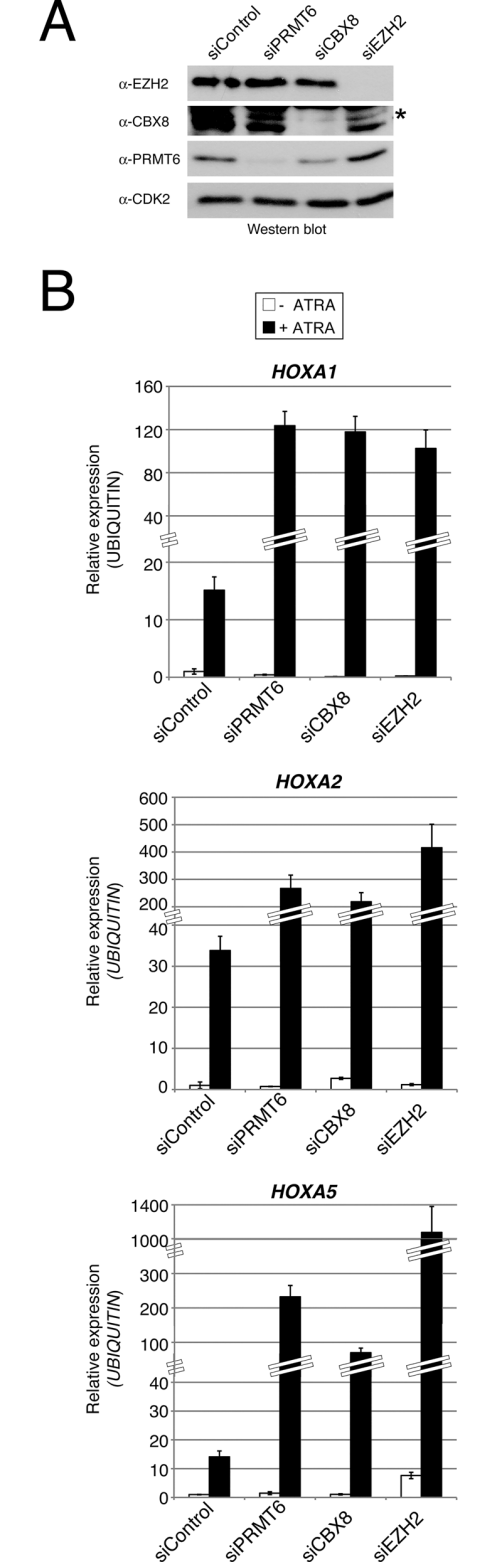
Rostral *HOXA* genes are common target genes of PRMT6- and PRC-mediated repression. (**A**) NT2/D1 cells were transfected with control siRNAs (siControl) or siRNAs directed against PRMT6 (siPRMT6 = equal mixture of siPRMT6_1 and siPRMT6_2), CBX8 (siCBX8) or EZH2 (siEZH2). Subsequently, cells were harvested and 30 μg total protein of each sample were analysed by Western blot with the indicated antibodies. The asterisk indicates the specific signals for the CBX8 protein. (**B**) NT2/D1 cells were transfected as in (A). Forty-eight hours post transfection cells were left untreated (-) or treated for 2 days (+) with 0.1 μM ATRA. Total RNA was then prepared and analysed in triplicates by RT-qPCR for transcript levels of *HOXA1*, *A2* and *A5* normalised to *UBIQUITIN* transcription. Error bars represent mean +/- S.D. of the triplicates. Transcript levels of untreated and siControl-transfected NT2/D1cells were set to 1.

We next asked whether PRC complexes, similar to PRMT6, are important for modulating the expression of *HOXA* genes during differentiation. Therefore, we additionally established siRNA-mediated depletion of CBX8 and EZH2 in NT2/D1 cells. An efficient knockdown was detected by Western blot analysis for all three proteins (Fig 3A). Similar to the effects upon PRMT6 depletion, RT-qPCR analysis revealed that depletion of CBX8 and EZH2 resulted in an increased transcriptional activation of *HOXA1*, *A2* and *A5* genes in response to ATRA compared to control transfected cells, while the basal transcript levels of the rostral *HOXA* genes remained unaltered in the absence of ATRA (Fig 3B). As observed upon PRMT6 depletion, the transcription of caudal *HOXA* genes, *HOXA9* and *A10*, was not significantly affected by depletion of CBX8 and EZH2, neither in undifferentiated nor in differentiated cells (data not shown). These results indicate that PRMT6, PRC1 (represented by the CBX8 subunit) and PRC2 (represented by the EZH2 subunit) function as direct transcriptional repressors of rostral *HOXA* genes to control their differentiation-associated activation in NT2/D1 cells. Although transcriptional activation of these target genes is accompanied by reduced PRMT6, PRC1 and PRC2 occupancy as well as H3R2me2a and H3K27me3 levels at the corresponding regulatory regions (Fig 1B), PRMT6- and Polycomb-dependent repression still seem to be responsible for constraining rostral *HOXA* gene activation. Our observations suggest that PRMT6 and PRC1/PRC2 are not required for maintaining the transcriptional repression of *HOXA* genes in the undifferentiated NT2/D1 cells, but rather confine their ATRA-induced gene activation.

### PRMT6 interacts with PRC1 and PRC2 subunits

**Fig 4:**
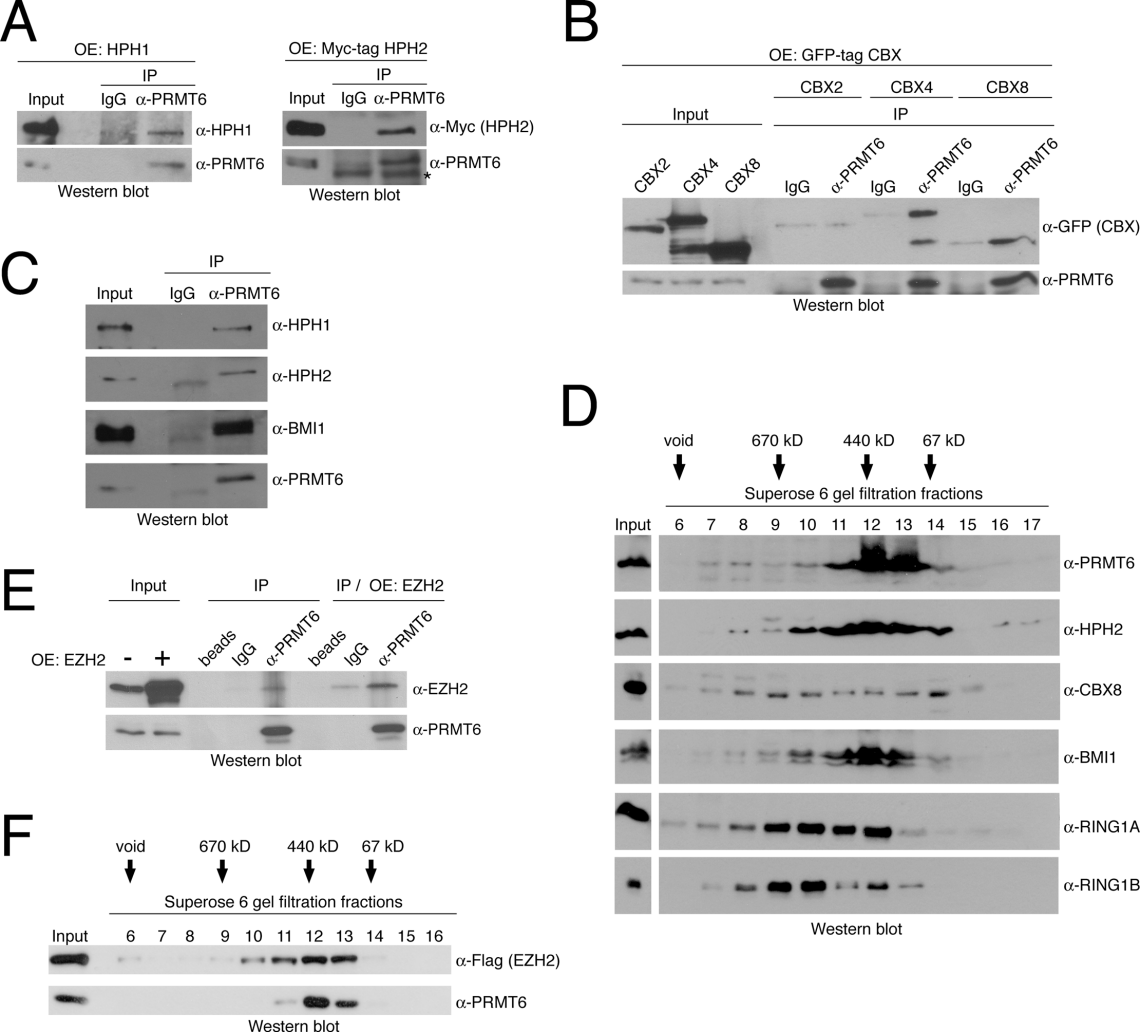
PRMT6 interacts with subunits of PRC1 and PRC2. (**A**) HEK293 cells were transfected with untagged HPH1 or Myc-tagged HPH2 constructs (OE = overexpressed) and harvested 48 hours after transfection. Protein extracts were subjected to immunoprecipitation using antibodies against PRMT6 (α-PRMT6) or isotype-specific IgG as control. Inputs (2%) and precipitates were subjected to Western blot analysis using antibodies against HPH1 (α-HPH1), Myc-tag (α-Myc for HPH2 detection) and PRMT6 (α-PRMT6). The asterisk indicates an unspecific signal in the α-PRMT6 staining. (**B**) HEK293 cells were transfected with GFP-tagged CBX2, CBX4 and CBX8 constructs (OE = overexpressed) and harvested 48 hours after transfection. Protein extracts were subjected to immunoprecipitation using antibodies against PRMT6 (α-PRMT6) or as control isotype-specific IgG. Input (2%) and precipitates were subjected to Western blot analysis using antibodies against GFP (α-GFP for CBX detection) and PRMT6 (α-PRMT6). (**C**) For the detection of endogenous interactions, HEK293 protein extracts were subjected to immunoprecipitation using antibodies against PRMT6 (α-PRMT6) or isotype-specific IgG as control. Input (2%) and precipitates were subjected to Western blot analysis using antibodies against HPH1 (α-HPH1), HPH2 (α-HPH2), BMI1 (α-BMI1) and PRMT6 (α-PRMT6). (**D**) For size fractionation by gel filtration chromatography, whole-cell protein extracts were prepared from HEK293 cells. Protein extracts were applied to a Superose 6 column and 6 ml fractions were collected. Five % of each fraction (no. 6–17) were analysed by Western blot using the indicated antibodies to detect PRMT6, HPH2, CBX8, BMI1, RING1A and RING1B, endogenously. The column was calibrated using standard protein markers. Accordingly, the molecular weight included in the fractions and the void volume (V_0_) are indicated. (**E**) For the detection of endogenous and exogenous interaction between PRMT6 and EZH2, HEK293 cells were either not transfected or transfected with Flag-tagged EZH2 construct (OE = overexpressed). Subsequently, protein extracts were subjected to immunoprecipitation using antibodies against PRMT6 (α-PRMT6) or isotype-specific IgG as control. Input (2%) and precipitates were analysed by Western blot using the indicated antibodies. (**F**) Whole-cell protein extracts were generated from HEK293 cells transfected with Flag-tagged EZH2 construct. Size fractionation by gel filtration chromatography was performed as described in (D) and fractions 6–16 were subjected to Western blot analysis using the indicated antibodies.

Given our observation that PRMT6 and H3R2me2a binding sites are as well occupied by PRCs and H3K27me3 at the *HOXA* cluster and given that the occurrence of both histone modifications correlates at silent gene promoters [28], we asked whether these enzymes and complex subunits physically interact. To address this question we performed CoIP (co-immunoprecipitation) assays to investigate protein-protein interactions between PRMT6 and subunits of PRC1 as well as PRC2 complexes. We initially overexpressed several core components of PRC1 complexes including the human PH homologues HPH1 and HPH2 as well as the CBX variants CBX2, CBX4 and CBX8. Immunoprecipitation of endogenous PRMT6 followed by Western blot staining to detect the exogenous PRC1 subunits revealed that HPH1, HPH2, CBX4 and CBX8 interact with PRMT6, whereas no binding was observed between PRMT6 and CBX2 (Fig 4A and 4B). We further confirmed the interactions between PRMT6 and the PH homologues on endogenous protein level (Fig 4C). In addition, CoIP-assays for the PSC homologues BMI1 and MEL18 indicated that endogenous BMI1 associates with PRMT6 (Fig 4C), whereas MEL18 showed no or only very weak interaction with the arginine methyltransferase (S2 Fig). The same results were obtained after addition of ethidium bromide in the IPs indicating that these interactions are not DNA-mediated (data not shown). To answer the question of whether the interaction with PRC1 components is a general characteristic of arginine methyltransferases, we performed similar analyses for PRMT1 and PRMT4. Thereby we demonstrated that these two PRMTs do not interact with BMI1 or HPH2 (S3 Fig) suggesting that the association with PRC1 subunits is a specific feature of PRMT6.

As several PRC1 complexes with alternate subunit composition have been identified in mammals [49,50], we next performed Superose 6 gel filtration analysis to determine whether PRMT6 associates with such PRC1-type complexes. We found that endogenous PRMT6 elutes in molecular weight fractions (maximum in fraction no. 12) of approximately 440 kD (Fig 4D, S4 Fig), which is a molecular weight higher than expected from its monomeric (42 kD) or dimeric size. Notably, PRMT6 co-eluted with endogenous (Fig 4D) and exogenous (S4 Fig) PRC1 components, such as HPH2, CBX8, BMI1, RING1A and RING1B. CoIP assays of the PRMT6-containing gel filtration fractions indicated that PRMT6 interacts with CBX8 and HPH2 in these high-molecular weight fractions (S5 Fig) suggesting that PRMT6 stably associates with the CBX-containing PRC1-type complex.

To examine whether PRMT6 interacts with PRC2 subunits, in particular EZH2, we transiently overexpressed Flag-tagged EZH2. As shown in Fig 4E, endogenous PRMT6 co-immunoprecipitated with exogenous as well as endogenous EZH2 (Fig 4E). Furthermore, gel filtration analysis showed that Flag-tagged EZH2 mainly co-elutes with PRMT6 in fraction no. 12, although EZH2 was also present in higher molecular weight fractions (no. 9 and 10) not containing PRMT6 (Fig 4F). We found that PRMT6 co-immunoprecipitates with EZH2 in such gel filtration fractions, in which both proteins co-eluted from the column (S6 Fig). Together these findings indicate that PRMT6, even though it does not seem to be an integral component of the core PRC complexes, transiently associates with PRC subunits as well as PRC complexes and thereby might influence the repressive capacity of these complexes.

### PRMT6 influences PRC-mediated gene silencing at the rostral *HOXA* genes locus

**Fig 5:**
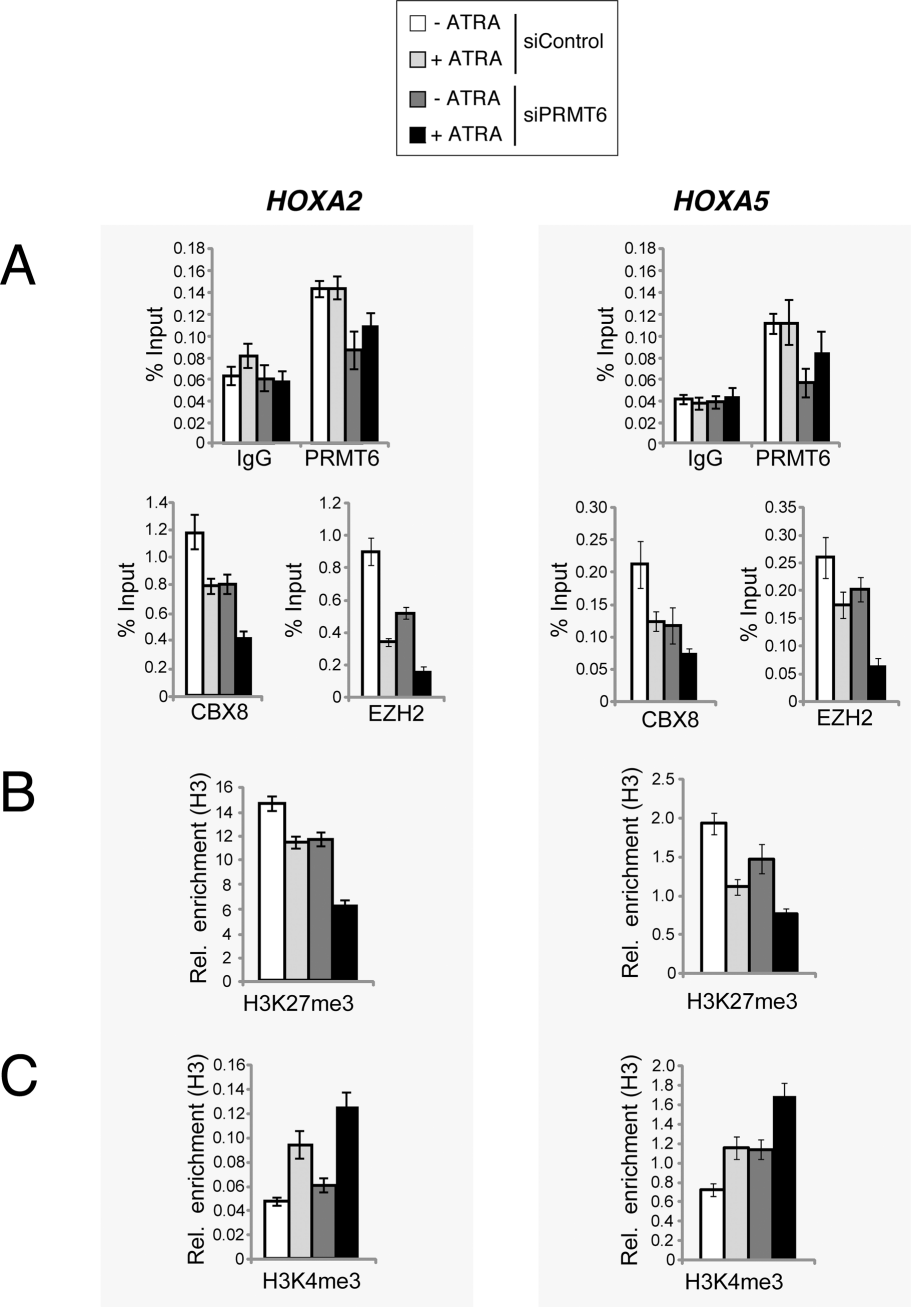
PRMT6 influences PRC-mediated gene silencing at the *HoxA* gene locus. (**A**) NT2/D1 cells were transfected with control siRNAs (siControl) or siRNAs directed against PRMT6 (siPRMT6 = equal mixture of siPRMT6_1 and siPRMT6_2). Forty-eight hours post transfection cells were left untreated (-) or treated for 2 days (+) with 0.1 μM ATRA. Subsequently, cells were subjected to ChIP analysis using PRMT6-, CBX8- as well as EZH2-specific antibodies. IgG served as control antibody for these three antibodies and is displayed together with the PRMT6 ChIP in the upper graph. Immunopreciptiated DNA was analysed in triplicates by qPCR with primers for the *HOXA2* and *A5* genes. Mean values are calculated as percentage input (% input) and error bars represent mean +/- S.D. of the triplicates. (**B, C**) NT2/D1 cells were transfected and treated as described in (A). Cells were then subjected to ChIP analysis using antibodies recognising histone H3 and the histone marks H3K27me3 (B) and H3K4me3 (C). Immunoprecipitated DNA was analysed in triplicates by qPCR with primers for the *HOXA2* and *A5* genes. Mean values are calculated as enrichment relative to histone H3 and error bars represent mean +/- S.D. of the triplicates.

As PRMT6 binding sites overlap with those of the PRC subunits CBX8 and EZH2 at regulatory regions within the *HOXA* gene locus and PRMT6 physically interacts with both proteins, we examined whether PRMT6 impacts Polycomb-dependent repression of the rostral *HOXA* genes. Therefore, we performed ChIP analysis of PRC subunits CBX8 and EZH2 as well as H3K27me3 at rostral *HOXA* genes in siPRMT6- or siControl-transfected NT2/D1 cells, which were either undifferentiated or ATRA-differentiated. The occupancy of PRMT6 was reduced at the *HOXA2* and *A5* gene promoters in the PRMT6-depleted conditions (Fig 5A). Interestingly, binding of CBX8 and EZH2 to the *HOXA2* and *A5* gene promoters was diminished upon depletion of PRMT6 both in the absence and presence of ATRA (Fig 5A). These events coincided with a decrease in H3K27me3 levels (Fig 5B) suggesting that PRMT6 positively influences the deposition of this mark, possibly via its association with the PRC1 complex and EZH2. To test the hypothesis that PRMT6-mediated deposition of H3R2me2a might directly promote the catalytic activity of EZH2, we performed *in vitro* methyltransferase assays using recombinant PRC2 complex in the presence of unmodified and premodified H3 peptides. To first validate the assay we determined the methylation activity of EZH2 towards unmodified and H3K27me3-premodified peptides. While a methylation product was detected by autoradiography for the unmodified H3 peptide, no signal was obtained when the H3K27me3-premodified peptide was employed as a substrate confirming that H3K27 is the major methylation site of recombinant PRC2 complex in our assay (S7 Fig). Premodification at R2 either by mono-methylation or asymmetric dimethylation did not enhance the activity of EZH2 arguing against a positive crosstalk at the level of these histone modifications (S7 Fig). In agreement with previous observations [5], we found that depletion of PRMT6 results in increased levels of H3K4me3 at the *HOXA2* and *A5* gene promoters in NT2/D1 cells (Fig 5C). As H3K4me3 has been reported to inhibit recognition of the H3 N-terminus by PRC2 and thereby the PRC2 activity [51], PRMT6 might promote the occurrence of H3K27me3 and Polycomb-mediated repression by constraining the levels of H3K4me3.

Together, these data uncover a novel and additional mechanism of how PRMT6 contributes to gene repression during cellular differentiation. Apart from counteracting the occurrence of H3K4me3, PRMT6 interacts with components of PRC1/PRC2 and facilitates chromatin binding of these subunits and the deposition of H3K27me3. Our observations suggest that PRMT6- and PRC1/PRC2-mediated repression mechanisms cooperate in fine-tuning and controlling appropriate timing and magnitude of ATRA-induced transcriptional activation of rostral *HOXA* genes.

## Discussion

The PRMT family member PRMT6 catalyses asymmetric dimethylation of histone H3 at arginine 2 and thereby causes transcriptional repression of PRMT6-bound genes by antagonising the deposition of the active histone mark H3K4me3 by the MLL1 complex [5,13,20,23]. This mechanism of transcriptional repression has been shown to be relevant during differentiation processes, such as PRMT6 acts as a co-repressor of the transcription factor RUNX1 thereby inhibiting megakaryocytic genes in hematopoietic progenitor cells and later the erythroid-specific gene expression program during megakaryocytic differentiation [29,30]. Furthermore, PRMT6 influences the expression of pluripotency and differentiation markers in embryonic stem cells [52]. Our own observations revealed that PRMT6 associates with differentiation-associated genes in neural progenitor cells [5], in line with high expression levels of PRMT6 in the developing mouse brain [53]. Therefore, we studied here the repressive role of PRMT6 and its functional relationship with neighbouring histone marks during neural differentiation using the EC model of NT2/D1 cells, which predominantly differentiate along the neuronal lineage upon exposure to ATRA.

H3R2me2a co-occurs at the genomic level with H3K27me3, a well-established repressive histone mark [28–30]. In addition, the presence of H3K27me3 and its writer as well as reader complexes (PRC2 and PRC1) have been described in detail at the *HOXA* gene cluster in NT2/D1 cells [36,48,54]. In undifferentiated cells, the *HOXA* gene locus is marked by high levels of H3K27me3 and bound by EZH2 (PRC2) as well as CBX8 (PRC1). Upon ATRA-induced neuronal differentiation, H3K27me3 and PRCs are specifically displaced from the rostral *HOXA* genes (*HOXA1-5*) in agreement with their transcriptional activation, whereas the caudal *HOXA* genes (*HOXA9-13*) keep high amounts of this repressive mark and the PRCs and unchanged expression levels [36,48,55]. Interestingly, PRMT6 and its histone mark H3R2me2a show a binding pattern at the *HOXA* cluster in undifferentiated and differentiating NT2/D1 cells similar to the one of H3K27me3 and the PRCs indicating that this locus is appropriate for studying a putative crosstalk between the PRMT6 and Polycomb proteins in target gene repression.

To elucidate the potential impact of PRMT6 as well as the PRCs on the transcriptional activity of the *HOXA* locus, we depleted the relevant enzymes and representative subunits of the complexes. We found that PRMT6 and PRC1/2 did not influence the expression of caudal *HOXA* genes, neither in undifferentiated nor in differentiating NT2/D1 cells. This is an agreement with previous observations showing that PRC1/2 binding does not strictly correlate with transcriptional silencing, but also occurs at transcriptionally active genes, which seems to hold true also for PRMT6 [36]. Furthermore, not all target genes of PRC1 and PRC2 are derepressed upon PRC depletion, but are thought to have acquired an additional, more permanent silencing, such as DNA methylation or higher order chromatin structure, which makes the stable presence of PRC1/2 dispensable [31,36]. However, we surprisingly observed that PRMT6 and PRC1/2 specifically restrict the transcriptional activation of rostral *HOXA* genes in differentiating NT2/D1 cells, although the occupancy of PRMT6, PRC1/2 as well as their histone marks was reduced at these gene promoters upon differentiation. This further supports the view that binding of PRMT6 and PRCs is diminished at differentiation-specific genes to enable their transcriptional induction, while they still seem to be required for constraining and fine-tuning the transcriptional response.

Given that PRMT6 and subunits of PRC1 and PRC2 coincide in their binding pattern at rostral *HOXA* genes and have a similar repressive influence on the ATRA-induced transcriptional activation of these genes, we investigated whether PRMT6 would directly regulate PRC-dependent repression. Depletion of PRMT6 resulted in reduced occupancy of CBX8 and EZH2 at *HOXA2* and *A5* gene promoters and in decreased levels of H3K27me3, independently of the differentiation status of the cells. In differentiating NT2/D1 cells these observations are in agreement with an enhanced ATRA-mediated transcriptional activation of *HOXA2* and *A5*, whereas the basal activity of these genes was not derepressed in undifferentiated cells. These results imply that PRMT6- and PRC-mediated repression cooperate in the transcriptional regulation of cell-fate genes. Although we found that PRMT6 is able to physically interact with several subunits of PRC1 and with EZH2, we hypothesise that PRMT6 is not responsible for the chromatin recruitment of PRCs via protein-protein interactions. We postulate that PRMT6 associates with PRC components at the chromatin and stimulates a histone modification landscape, such as increased H3R2me2a levels and decreased H3K4me3 levels, in the vicinity of PRC binding sites, which stabilises PRC binding and enhances the catalytic activity of EZH2. In agreement with this model, our data indicate that PRMT6 is not a stable component of core PRC complexes, but rather support that PRMT6 transiently associates with PRC subunits and complexes to regulate their repressive capacity.

Given the complex interplay between different histone modifications and the fact that a preexisting modification can promote or inhibit the subsequent occurrence of another histone mark, such as H3R2me2a counteracting the deposition of H3K4me3 (5, 13), we asked whether the positive crosstalk between PRMT6 and PRCs is mediated via the H3R2me2a mark. Therefore, we assayed the catalytic activity of EZH2 on unmodified as well as H3R2me2a-premodified H3 peptides. Monomethylation or asymmetric dimethylation at R2 did not enhance the activity of EZH2 indicating that PRMT6 does not directly influence the EZH2 activity via depositioning the H3R2me2a mark. However, H3K4me3 levels were increased at the *HOXA2* as well as *A5* gene promoter in PRMT6-depleted NT2/D1 cells, in particular upon ATRA treatment. As H3K4me3 has been reported to inhibit the recognition of histone H3 N-terminus by PRC2 and thereby the PRC2 activity [51], we hypothesise that PRMT6 promotes the occurrence of H3K27me3 and consequently the recruitment of CBX8 at rostral *HOXA* genes in an indirect manner via associating with PRC complexes and thereby restricting the occurrence and magnitude of H3K4me3.

In summary, we postulate a model, in which neither PRMT6 nor PRCs are necessary for maintaining the transcriptional status (low basal activity) of the *HOXA* gene cluster in undifferentiated NT2/D1 cells despite their presence at the chromatin. This situation also applies to the caudal *HOXA* genes in differentiating cells suggesting that here additional, more permanent silencing mechanisms have been acquired. However, upon ATRA treatment rostral *HOXA* genes are transcriptionally activated, which is facilitated by an increase in H3K4me3 levels via MLL1 complex recruitment with the concomitant reduction of PRMT6 and PRC1/2 recruitment and their corresponding histone marks. Even during this transcriptional activation a balance of activating and silencing mechanisms seem to be necessary to enable an adequate transcriptional response, since PRMT6 and PRCs are important for constraining the ATRA-mediated transcriptional response of rostral *HOXA* genes. Altogether, our results confirm the previously reported countercorrelation between PRMT6 and MLL1 activities and uncover a novel cooperation between PRMT6- and PRC-mediated repression.

## Supporting Information

S1 Fig Alternative siRNAs targeting PRMT6 confirm the function of PRMT6 in repressing the ATRA-mediated transcriptional activation of rostral *HOXA* genes. NT2/D1 cells were transfected with control siRNAs (siControl) or 8 alternative siRNAs directed against PRMT6 (including the mixture of siPRMT6_1 and siPRMT6_2, which was used in Fig 3 and Fig 5). Forty-eight hours post transfection cells were left untreated (-) or treated for 2 days (+) with 0.1 μM ATRA. Subsequently, total RNA was prepared and analysed in triplicates by RT-qPCR for transcript levels of *PRMT6*, *HOXA1*, *A2* and *A5* normalised for *UBIQUITIN* transcription. Error bars represent mean +/- S.D. of the triplicates. Transcript levels of untreated and siControl-transfected NT2/D1 cells were set to 1.
(TIF)

S2 Fig PRMT6 does not or only weakly interact with MEL18. HEK293 cells were transfected with GFP-tagged MEL18 construct and harvested 48 hours after transfection. Protein extracts were subjected to immunoprecipitation using antibodies for PRMT6 (α-PRMT6) or as controls beads alone as well as isotype-specific IgG. Input (2%) and precipitates were subjected to Western blot analysis using antibodies against GFP (α-GFP for MEL18 detection) and PRMT6 (α-PRMT6).
(TIF)

S3 Fig PRMT1 and PRMT4 do not interact with subunits of the PRC1 complex, such as BMI1 and HPH2. HEK293 protein extracts were subjected to immunoprecipitation using antibodies for PRMT1 (α-PRMT1), PRMT4 (α-PRMT4) or as controls beads alone as well as isotype-specific IgG. Input (2%) and precipitates were subjected to Western blot analysis using antibodies against BMI1 (α-BMI1), HPH2 (α-HPH2), PRMT1 (α-PRMT1) and PRMT4 (α-PRMT4). The asterisk indicates the specific signals for the PRMT4 protein.
(TIF)

S4 Fig PRMT6 co-elutes with the PRC1 subunits HPH2 and CBX8 in the same high molecular weight fractions. For size fractionation by gel filtration chromatography, HEK293 cells were transfected with GFP-tagged CBX8 and Myc-tagged HPH2 constructs. Subsequently whole-cell protein extracts were applied to a Superose 6 column and 6 ml fractions were collected. Five % of each fraction (no. 6–15) were analysed by Western blot using the indicated antibodies to detect GFP (α-GFP for CBX8 detection), Myc-tag (α-Myc for HPH2 detection) and PRMT6 (α-PRMT6). The column was calibrated using standard protein markers. Accordingly, the molecular weight included in the fractions and the void volume (V_0_) are indicated.
(TIF)

S5 Fig PRMT6 stably interacts with CBX8 and HPH2 in co-elution gel filtration fractions. HEK293 cells were transfected with GFP-tagged CBX8 and Myc-tagged HPH2 constructs. Whole-cell protein extracts (WCE) and the corresponding Superose 6 gel filtration fractions (no. 6–15) were subjected to immunoprecipitation using antibodies against PRMT6 (α-P6, α-PRMT6) or as control isotype-specific IgG (only for WCE). Input (2%) of WCE and precipitates were analysed by Western blot using antibodies against GFP (α-GFP for CBX8 detection), Myc-tag (α-Myc for HPH2 detection) and PRMT6 (α-PRMT6).
(TIF)

S6 Fig PRMT6 stably interacts with EZH2 in co-elution gel filtration fractions. HEK293 cells were transfected with Flag-tagged EZH2 construct. Whole-cell protein extract (WCE) and the corresponding Superose 6 gel filtration fractions (no. 6–16) were subjected to IP using antibodies against PRMT6 (α-P6, α-PRMT6) or as control isotype-specific IgG (only for WCE). Input (2%) of WCE and precipitates were subjected to Western blot analysis using antibodies against Flag-tag (α-Flag for EZH2 detection) and PRMT6 (α-PRMT6).
(TIF)

S7 Fig The catalytic activity of recombinant PRC2 is not enhanced by R2me2a-premodification of H3. Recombinant PRC2 complex (400 ng) and 4 μg of either unmodified, R2me1, R2me2a or K27me3 premodified H3 peptides (aa 1–30) were incubated in the presence or absence of [^14^C-methyl]-SAM overnight at 30°C. Subsequently, the methyltransferase reactions were analysed by SDS-PAGE, blotting and autoradiography. Ponceau S staining of the blot was used as loading control for the different H3 peptides.
(TIF)

S1 Text Supporting Information Materials and Methods. (DOCX)
