# Supplementary figures and images for: The Arginine Methyltransferase PRMT6 Cooperates with Polycomb Proteins in Regulating HOXA Gene Expression
Source: PLoS One. 2016 Feb 5;11(2):e0148892. doi: 10.1371/journal.pone.0148892 (PMC4746130; doi:10.1371/journal.pone.0148892)

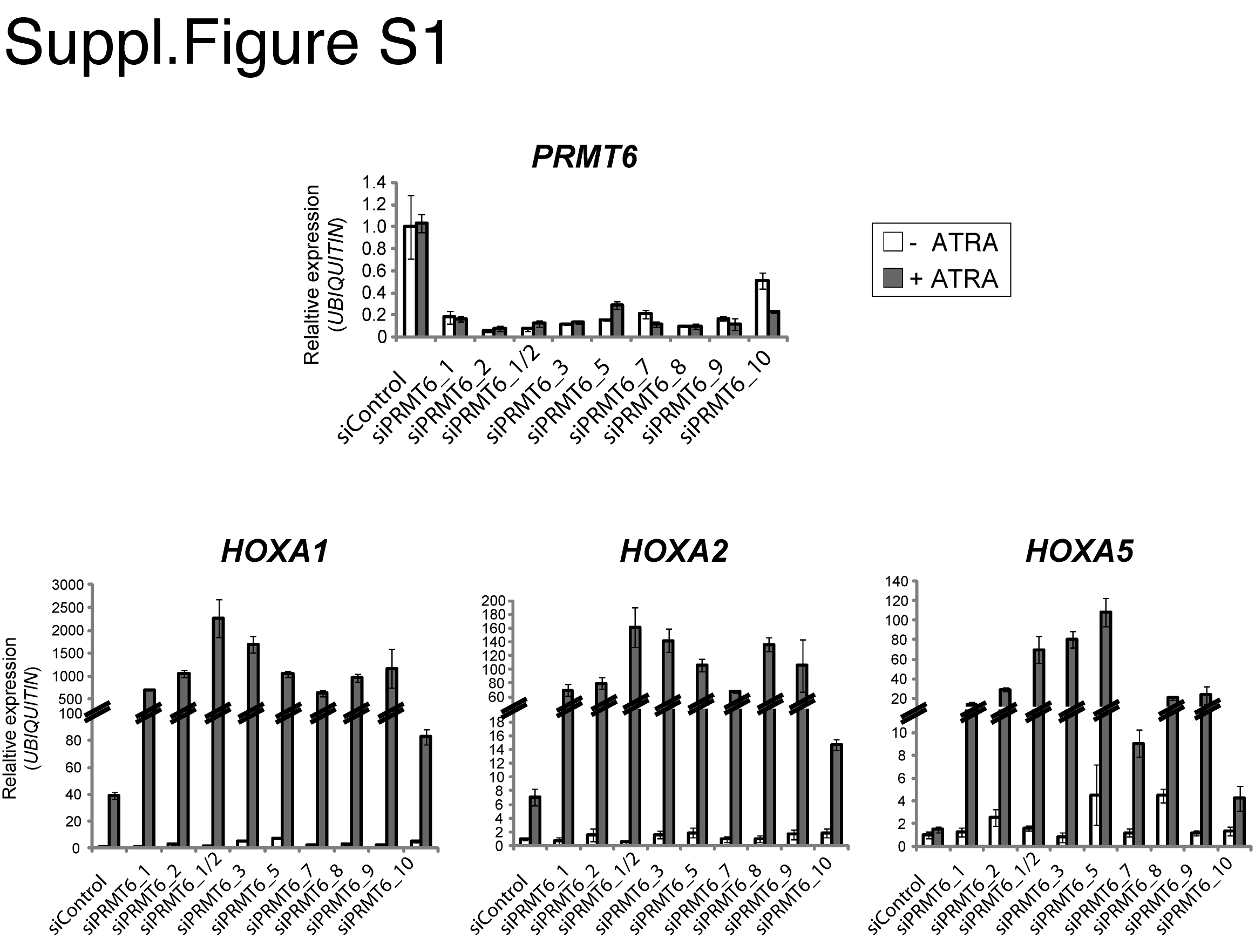

Supplement: S1 Fig — NT2/D1 cells were transfected with control siRNAs (siControl) or 8 alternative siRNAs directed against PRMT6 (including the mixture of siPRMT6_1 and siPRMT6_2, which was used in Fig 3 and Fig 5). Forty-eight hours post transfection cells were left untreated (-) or treated for 2 days (+) with 0.1 μM ATRA. Subsequently, total RNA was prepared and analysed in triplicates by RT-qPCR for transcript levels of PRMT6, HOXA1, A2 and A5 normalised for UBIQUITIN transcription. Error bars represent mean +/- S.D. of the triplicates. Transcript levels of untreated and siControl-transfected NT2/D1 cells were set to 1. (TIF) [file pone.0148892.s001.tif]

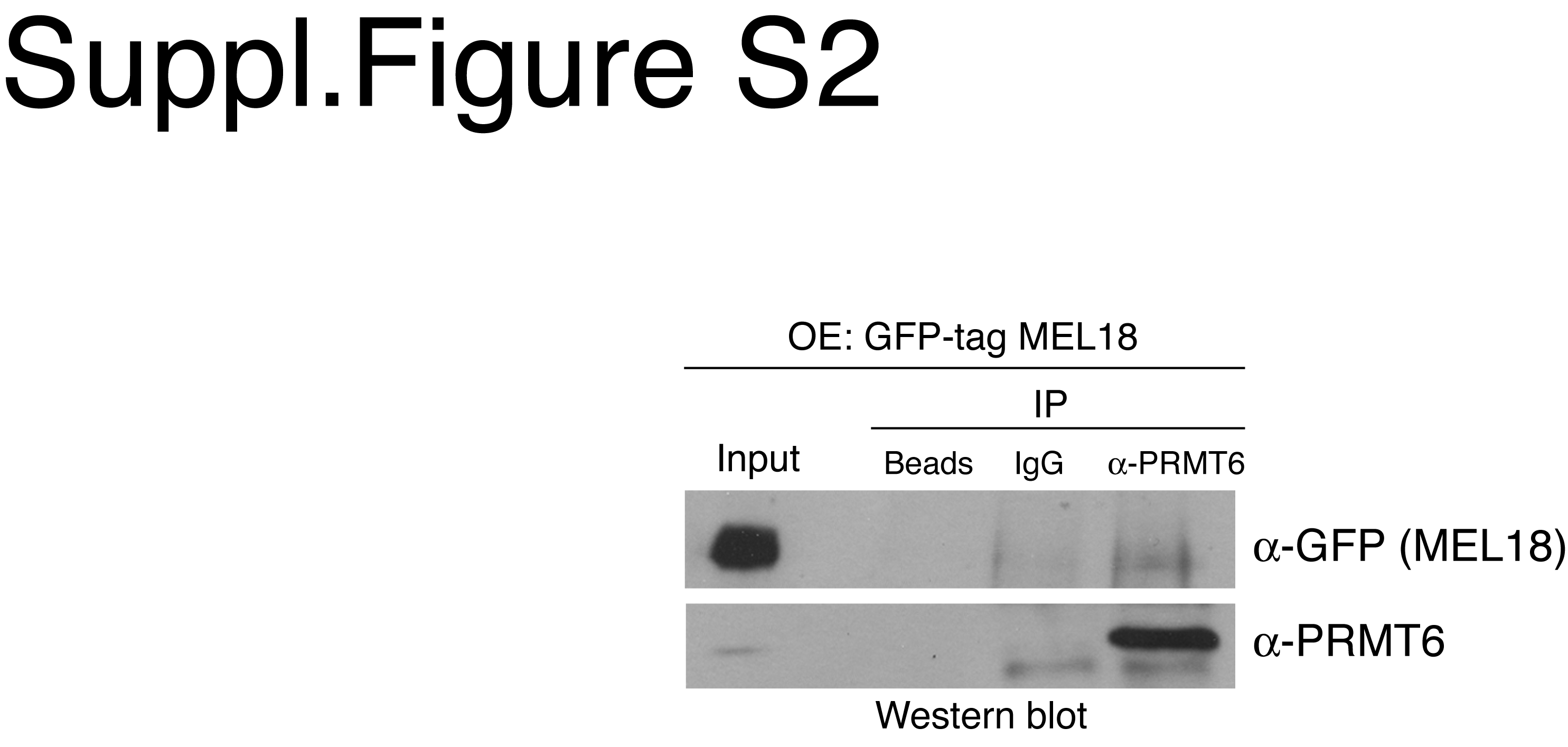

Supplement: S2 Fig — HEK293 cells were transfected with GFP-tagged MEL18 construct and harvested 48 hours after transfection. Protein extracts were subjected to immunoprecipitation using antibodies for PRMT6 (α-PRMT6) or as controls beads alone as well as isotype-specific IgG. Input (2%) and precipitates were subjected to Western blot analysis using antibodies against GFP (α-GFP for MEL18 detection) and PRMT6 (α-PRMT6). (TIF) [file pone.0148892.s002.tif]

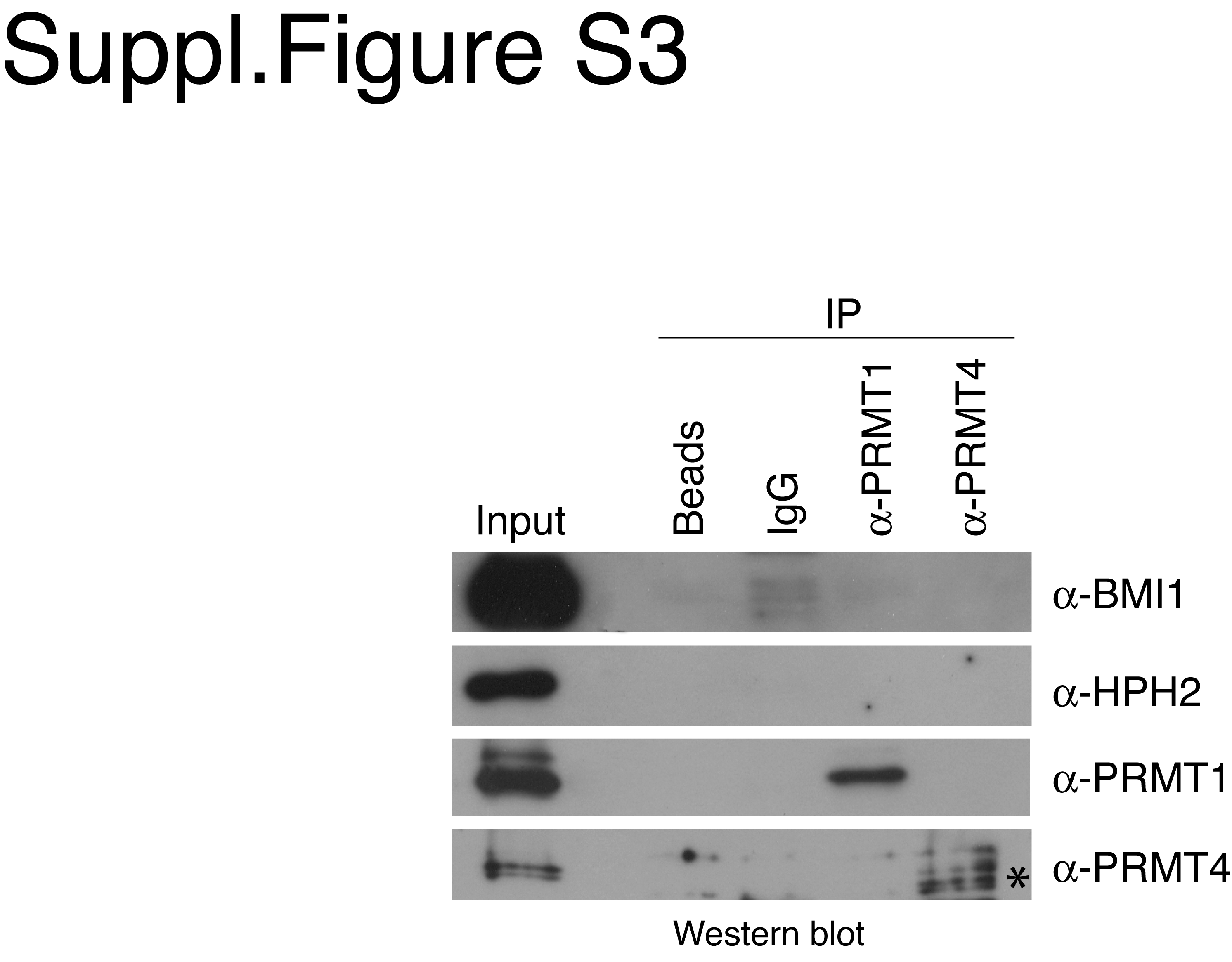

Supplement: S3 Fig — HEK293 protein extracts were subjected to immunoprecipitation using antibodies for PRMT1 (α-PRMT1), PRMT4 (α-PRMT4) or as controls beads alone as well as isotype-specific IgG. Input (2%) and precipitates were subjected to Western blot analysis using antibodies against BMI1 (α-BMI1), HPH2 (α-HPH2), PRMT1 (α-PRMT1) and PRMT4 (α-PRMT4). The asterisk indicates the specific signals for the PRMT4 protein. (TIF) [file pone.0148892.s003.tif]

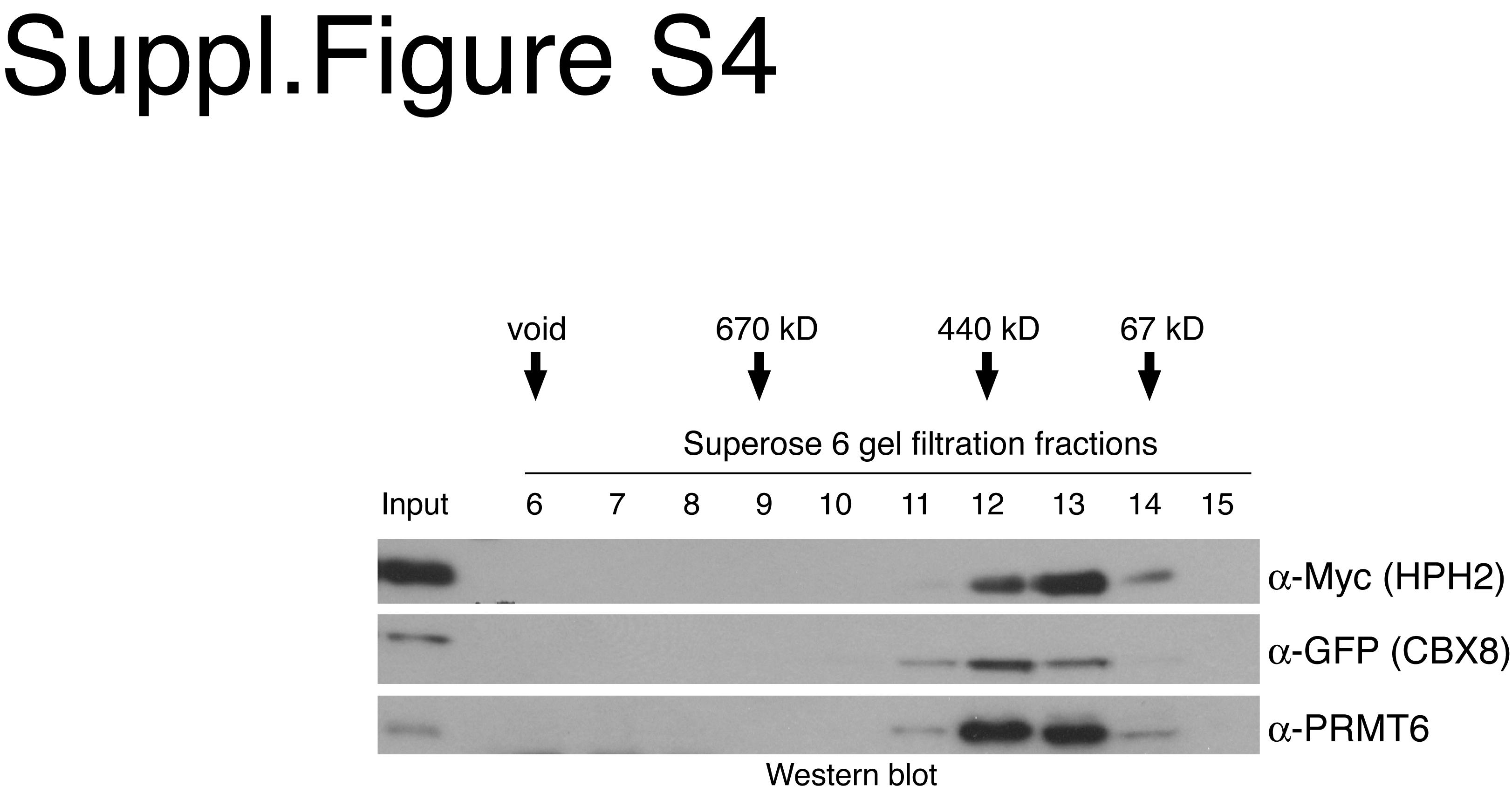

Supplement: S4 Fig — For size fractionation by gel filtration chromatography, HEK293 cells were transfected with GFP-tagged CBX8 and Myc-tagged HPH2 constructs. Subsequently whole-cell protein extracts were applied to a Superose 6 column and 6 ml fractions were collected. Five % of each fraction (no. 6–15) were analysed by Western blot using the indicated antibodies to detect GFP (α-GFP for CBX8 detection), Myc-tag (α-Myc for HPH2 detection) and PRMT6 (α-PRMT6). The column was calibrated using standard protein markers. Accordingly, the molecular weight included in the fractions and the void volume (V0) are indicated. (TIF) [file pone.0148892.s004.tif]

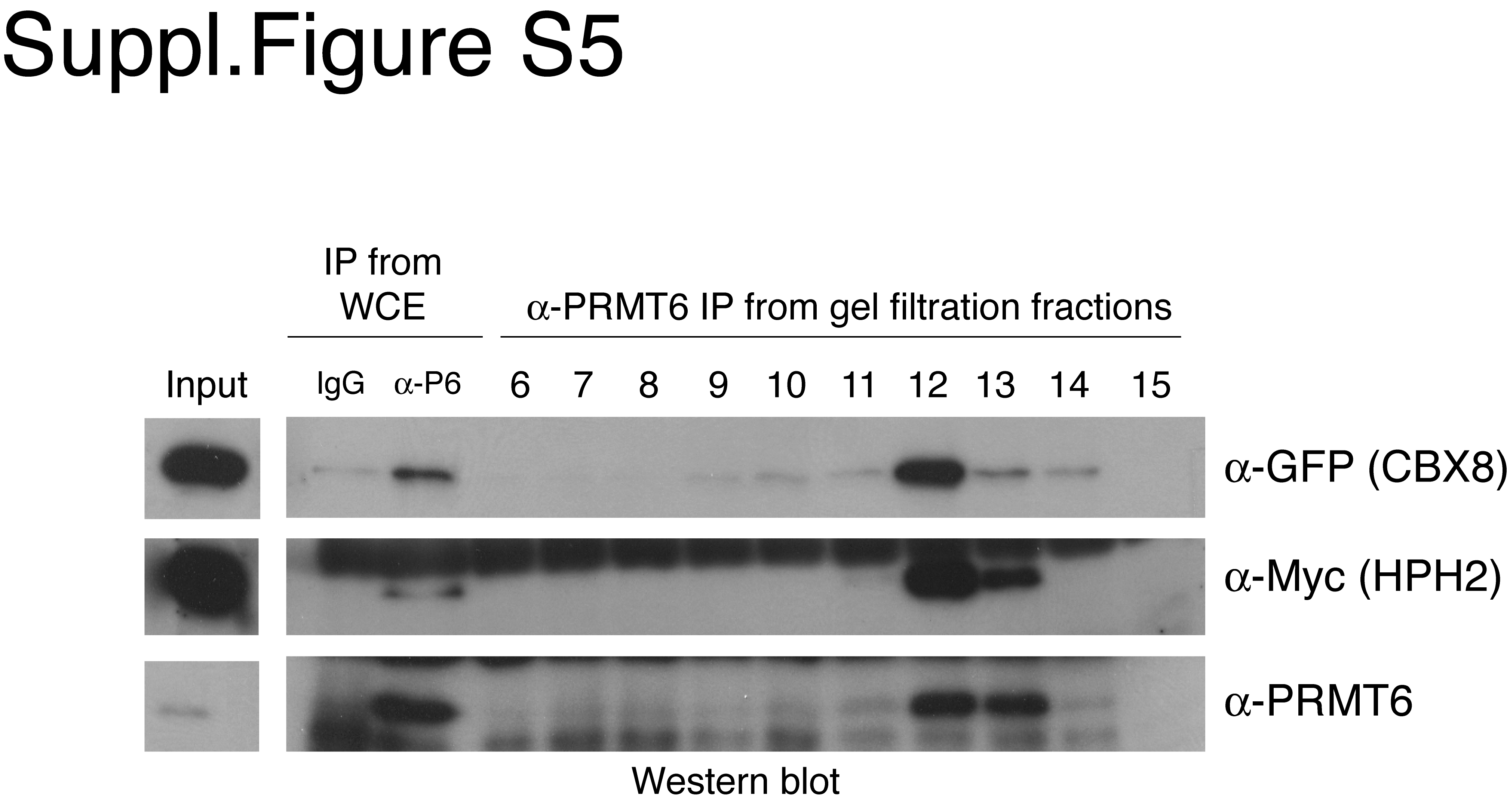

Supplement: S5 Fig — HEK293 cells were transfected with GFP-tagged CBX8 and Myc-tagged HPH2 constructs. Whole-cell protein extracts (WCE) and the corresponding Superose 6 gel filtration fractions (no. 6–15) were subjected to immunoprecipitation using antibodies against PRMT6 (α-P6, α-PRMT6) or as control isotype-specific IgG (only for WCE). Input (2%) of WCE and precipitates were analysed by Western blot using antibodies against GFP (α-GFP for CBX8 detection), Myc-tag (α-Myc for HPH2 detection) and PRMT6 (α-PRMT6). (TIF) [file pone.0148892.s005.tif]

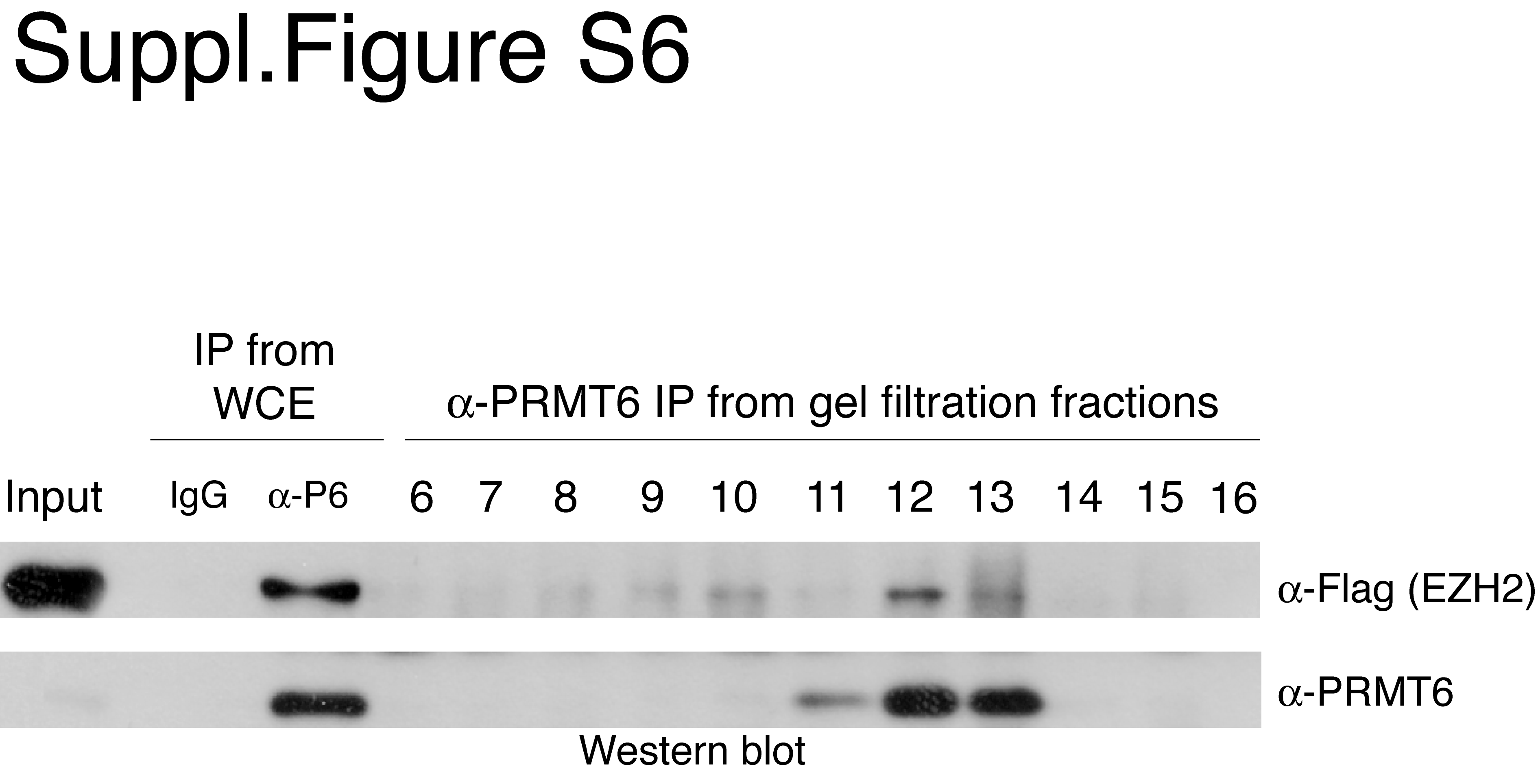

Supplement: S6 Fig — HEK293 cells were transfected with Flag-tagged EZH2 construct. Whole-cell protein extract (WCE) and the corresponding Superose 6 gel filtration fractions (no. 6–16) were subjected to IP using antibodies against PRMT6 (α-P6, α-PRMT6) or as control isotype-specific IgG (only for WCE). Input (2%) of WCE and precipitates were subjected to Western blot analysis using antibodies against Flag-tag (α-Flag for EZH2 detection) and PRMT6 (α-PRMT6). (TIF) [file pone.0148892.s006.tif]

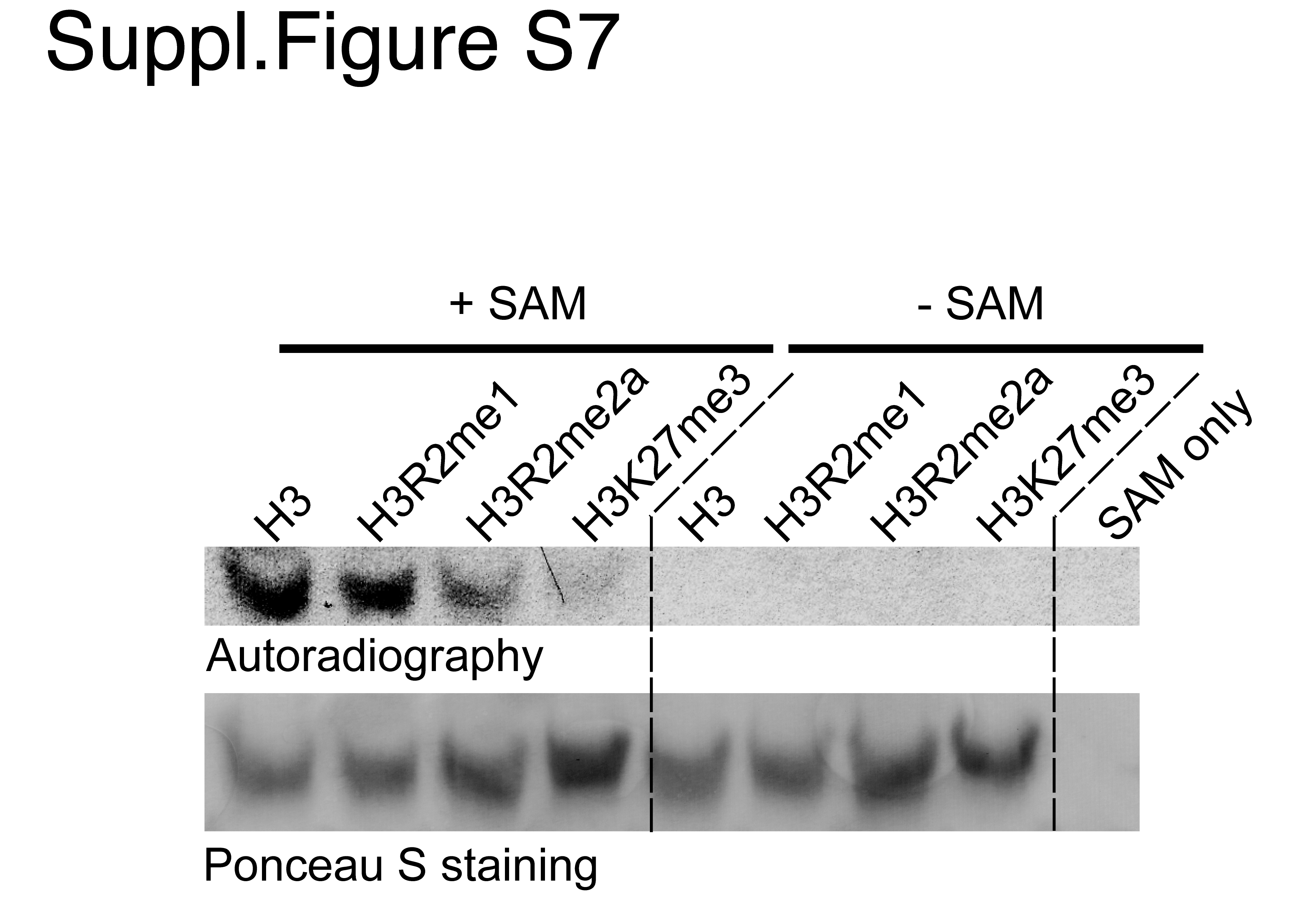

Supplement: S7 Fig — Recombinant PRC2 complex (400 ng) and 4 μg of either unmodified, R2me1, R2me2a or K27me3 premodified H3 peptides (aa 1–30) were incubated in the presence or absence of [14C-methyl]-SAM overnight at 30°C. Subsequently, the methyltransferase reactions were analysed by SDS-PAGE, blotting and autoradiography. Ponceau S staining of the blot was used as loading control for the different H3 peptides. (TIF) [file pone.0148892.s007.tif]
