# Supplementary material for: The Arginine Methyltransferase PRMT6 Cooperates with Polycomb Proteins in Regulating HOXA Gene Expression
Source: PLoS One. 2016 Feb 5;11(2):e0148892. doi: 10.1371/journal.pone.0148892 (PMC4746130; doi:10.1371/journal.pone.0148892)
Supplement: S1 Text — (DOCX) [file pone.0148892.s008.docx]

**Supporting Information Material and Methods (S1 Text)**

**Antibodies and plasmids**

The following antibodies and plasmids were employed for the Suppl.Figures: anti-PRMT1 from Millipore/Upstate (07-404) and anti-PRMT4 [1]. EGFP-MEL18 is described in [2].

**SiRNA sequences**

The sense strands of the siRNA sequences are indicated below. The siControl contained an equal mixture of three siRNAs (siNON, siLuciferase and siScrambled) and the siEZH2 contained an equal mixture of the three indicated siRNAs.

siNON 5’-UAAGGCUAUGAAGAGAUAC-3’

siLuciferase 5’-GAUUAUGUCCGGUUAUGUA-3’

siScrambled 5'-CAUAAGCUGAGAUACUUCA-3’

siPRMT6_1 5’-GAGCAAGACACGGACGUUU-3’

siPRMT6_2 5’-GCAAGACACGGACGUUUCA-3’

siPRMT6_3 5'-CUGUAGAGUUGCCGGAACA-3’

siPRMT6_5 5’-GGAGGGAGAGUGACUUCAU-3’

siPRMT6_7 5’-CAGCUGUACUACGAGUGCU-3’

siPRMT6_8 5'-GACGUUUCAGGAGAGAUCA-3’

siPRMT6_9 5’-CGGAACAGGUGGAUGCCAU-3’

siPRMT6_10 5’-GCUUUGCUCAGCUAGAGCU-3’

siCBX8 5’-GUACCAGCAGAGUGGAUGA-3’

siEZH2_2 5’-GAGGACGGCUUCCCAAUAA-3’

siEZH2_3 5’-GCUGAAGCCUCAAUGUUUA-3’

siEZH2_4 5’-GAAUGGAAACAGCGAAGGA-3’

**Primer sequences used in RT-qPCR and ChIP-qPCR**

For RT-qPCR, the following primers were used:

*HOXA1* forward 5’-CAGCCCCTACGCGTTAAAT-3’

reverse 5’-GGAGAAGATGTCTCCGATGC-3’

*HOXA2* forward 5’-TCTGGAGAGGGAAGGCTACA-3’

reverse 5’-TCGCCATTGTGTCCATTG-3’

*HOXA5* forward 5’-GCGCAAGCTGCACATAAG-3’

reverse 5’-CGGTTGAAGTGGAACTCCTT-3’

*HOXA9* forward 5’-ACACTATGAAACCGCCATTGG-3’

reverse 5’-GGAAACCCCAGATTCATCAAGG-3’

*HOXA10* forward 5’-CCACCTCACGGACAGACAAGTG-3’

reverse 5’-TTTCTTCAGTTTCATCCTGCGG-3’

*PRMT6* forward 5’-AGACACGGACGTTTCAGGAG-3’

reverse 5’-CCACTTTGTAGCGCAGCAG-3’

*UBIQUTIN* forward 5’-CACTTGGTCCTGCGCTTGA-3’

reverse 5’-CAATTGGGAATGCAACAACTTTAT-3’

For ChIP-qPCR, the following primers were used:

*HOXA1* RARE forward 5’-TCTTGCTTTGGCTCTGAAGTCT-3’

reverse 5’-GAGCTCAGATAAACTGCTGGGACT-3’

*HOXA2* promoter forward 5’-GAACTTATGTGGCTGGGACGCAG-3’

reverse 5’-GGACTGTCGGGAAGACGCCT-3’

*HOXA5* promoter forward 5’-CTCCACCCAACTCCCCTATTAGTG-3’

reverse 5’-GTGCGTCTATAGCACCCTTGC-3’

*HOXA9* promoter forward 5’-CCTGTGTGGCTTCTGAAACA-3’

reverse 5’-CAAATCGCATTGTCGCTCTA-3’

*HOXA10* promoter forward 5’-CGCCAAATTATCCCACAACAATGTC-3’

reverse 5’-CCCGAGCTGATGAGCGAGTC-3’

***In vitro* methyltransferase assay**

Recombinant PRC2 complex (400 ng, Active Motif) and 4 µg H3 peptides (aa 1-30) were incubated with 400 nCi [^14^C-methyl]-SAM (Bio Trend 2, 0.1 mCi/ml) in methyltransferase reaction buffer (5 mM MgCl, 4 mM DTT, 50 mM Tris, pH 8.0) overnight at 30°C. Subsequently, methyltransferase reactions were analysed by SDS-PAGE, blotting and autoradiography.

**References of the Supporting Information S1 Text**

1. Streubel G, Bouchard C, Berberich H, Zeller MS, Teichmann S, Adamkiewicz J, et al. PRMT4 is a novel coactivator of c-Myb-dependent transcription in haematopoietic cell lines. PLoS Genet. 2013;9: e1003343. Available: http://www.ncbi.nlm.nih.gov/entrez/query.fcgi?cmd=Retrieve&db=PubMed&dopt=Citation&list_uids=23505388

2. Zhang J, Goodson ML, Hong Y, Sarge KD. MEL-18 interacts with HSF2 and the SUMO E2 UBC9 to inhibit HSF2 sumoylation. J Biol Chem. 2008;283: 7464–7469. Available: http://www.ncbi.nlm.nih.gov/entrez/query.fcgi?cmd=Retrieve&db=PubMed&dopt=Citation&list_uids=18211895
